# Microwave-Assisted Synthesis of Few-Layer Ti_3_C_2_T_x_ Loaded with Ni_0.5_Co_0.5_Se_2_ Nanospheres for High-Performance Supercapacitors

**DOI:** 10.3390/ma17102292

**Published:** 2024-05-12

**Authors:** Linghong Wu, Juan Shen, Bo Jin

**Affiliations:** 1 School of Materials and Chemistry, Southwest University of Science and Technology, Mianyang 621010, China; wlh160130@163.com; 2 State Key Laboratory of Environmental-Friendly Energy Materials, Southwest University of Science and Technology, Mianyang 621010, China; jinbo0428@163.com

**Keywords:** microwave, few-layer Ti_3_C_2_T_x_, Ni_0.5_Co_0.5_Se_2_, battery type, asymmetric supercapacitors

## Abstract

Transition metal selenides have high theoretical capacities, making them attractive candidates for energy storage applications. Here, using the microwave-absorbing properties of the materials, we designed a simple and efficient microwave-assisted synthesis method to produce a composite made of nanospheres Ni_0.5_Co_0.5_Se_2_ (NCSe) and highly conductive, stable Ti_3_C_2_T_x_ MXene. The Ni_0.5_Co_0.5_Se_2_/Ti_3_C_2_T_x_ composites are characterized via scanning electron microscopy, X-ray diffraction, cyclic voltammetry, and electrochemical impedance spectroscopy. The findings indicate that 3D Ni_0.5_Co_0.5_Se_2_ bimetallic selenide nanospheres were uniformly loaded within the few-layer Ti_3_C_2_T_x_ MXene wrapper in a short period. The optimal NCSe/Ti_3_C_2_T_x_−2 electrode can demonstrate a specific capacitance of 752.4 F g^–1^ at 1 A g^–1^. Furthermore, the asymmetric supercapacitor combined with activated carbon maintains a capacitance retention of 110% even after 5000 cycles. The method of directly growing active substances on few-layer Ti_3_C_2_T_x_ MXene will provide inspiration for the manufacture of high-pseudocapacitance supercapacitors.

## 1. Introduction

Humankind has been continuously searching for energy sources, and a series of environmental problems brought about by the excessive exploitation and utilization of fossil fuels causes the demand for clean energy to increase each day [1,2]. Therefore, the development of high-power, inexpensive, safe, and stable energy storage devices to collect and store unstable clean energy is the basis for the large-scale application of renewable energy [3,4]. Supercapacitors have garnered extensive interest and secured a prominent role in the realm of energy storage within this societal context. This is due to their superior characteristics such as high power density, rapid charging and discharging capabilities, and exceptional safety performance [5,6,7].

Monometallic selenides like NiSe_2_, MoSe_2_, and CoSe_2_ are widely applied as electrode materials due to their high electrical conductivity and high theoretical specific capacitance, rendering them highly promising pseudocapacitive materials [8,9,10]. However, practical application remains challenging as it is difficult to prepare monometallic selenides with simultaneously high specific capacitance, excellent rate capability, and admirable cycling stability. The inherent synergy among bimetallic components enables these components to provide richer multi-electron redox reactions than monometallic components [11]. Nickel cobalt selenide (NiCoSe) is highly regarded as a prospective electrode material owing to its ample redox reaction sites and impressive theoretical capacity [12]. However, its low conductivity and stability limit further improvement of its electrochemical performance. An effective way to improve the electrochemical performance of NiCoSe is to combine NiCoSe with other suitable electrode materials to form a composite. Zhang et al. achieved the formation of a conductive 3D network by cultivating nanosheets of carbonyl sulfate (CoS) on the surface of NiCoSe nanotubes. Compared with the single-phase NiCoSe nanotube arrays, the NiCoSe@CoS nanotube arrays as electrodes demonstrated enhanced areal capacitance [13]. Jiang et al. used ultrathin Ti_3_C_2_T_x_ nanosheets wrapped with NiSe_2_ octahedral crystals to synthesize innovative NiSe_2_/Ti_3_C_2_T_x_ hybrid materials with strong interfacial interactions and electrochemical performance. The Ti_3_C_2_T_x_ nanosheets covered the surface of NiSe_2_ nanocrystals, hindering their oxidation and providing protection [14].

The two-dimensional (2D) transition metal carbides and/or nitrides (M_n+1_X_n_T_x_), called MXenes, exhibit elevated electrical conductivity, expansive specific surface area, superior mechanical strength, and favorable stability [15]. And the delaminated nature of MXene layers allows for efficient charge transport, contributing to their conductivity. Ti_3_C_2_T_x_, the first reported MXene, exhibits very excellent cycling performance and superior rate performance [16]. The inherent layer restacking of MXenes into a dense structure occurs during the electrode preparation process due to van der Waals interactions [17,18]. To solve these problems, we designed a structure by uniformly loading 3D Ni_0.5_Co_0.5_Se_2_ bimetallic selenide nanospheres within a Ti_3_C_2_T_X_ MXene wrapper.

Common electrode material synthesis methods include hydrothermal, precipitation, and sol–gel methods [19,20,21]. However, these methods take a long time, and the subsequent processing steps are cumbersome. Microwave reaction can release ultrahigh microwave energy in the instant to improve reaction kinetics. This process provides a highly efficient and energy conservation synthesis method [22].

Both selenide and few-layer Ti_3_C_2_T_X_ MXene exhibit strong microwave-absorbing properties, allowing for the efficient absorption of microwave energy. Therefore, we were able to achieve the growth of selenide in the structure of few-layer MXene in a short time. The aim of this study was to synthesize composites of Ni_0.5_Co_0.5_Se_2_ and Ti_3_C_2_T_x_ by one-step microwave synthesis and to investigate the electrochemical properties of the composites as a function of the addition of Ti_3_C_2_T_x_ and reaction time. The energy properties, power properties, and cycling stability of asymmetric supercapacitors containing Ni_0.5_Co_0.5_Se_2_/Ti_3_C_2_T_x_ as the active cathode material and activated carbon as the anode were also investigated. The microwave-assisted synthesis method enables the direct growth of selenide within the few-layer Ti_3_C_2_T_X_ MXene structure, potentially providing new insights into the preparation of high-pseudocapacitance supercapacitors.

## 2. Experimental Section

### 2.1. Materials

Ti_3_AlC_2_ powder (purity > 99%, 400 mesh) was commercially bought from Jilin 11 Technology Co., Ltd., Changchun, China. Lithium fluoride (LiF), activated carbon (AC), and selenium powder were purchased from Aladdin Chemistry Co., Ltd., Shanghai, China. Nickel acetate tetrahydrate [Ni(CH_3_COO)_2_·4H_2_O], cobalt acetate tetrahydrate [Co(CH_3_COO)_2_·4H_2_O], potassium hydroxide (KOH), hydrochloric acid (HCl), ethanediamine (C_2_H_8_N_2_), and ethanediol (C_2_H_6_O_2_) were obtained from Chengdu Colon Chemical Co., Ltd., Chengdu, China.

### 2.2. Preparation of Ti_3_C_2_T_x_

Ti_3_C_2_T_x_ was synthesized via an in situ etching method and ultrasonic exfoliation. Initially, 1.0 g of Ti_3_AlC_2_ MAX-phase precursor powder was gradually introduced into the etching solution, which consisted of 1.32 g of LiF dissolved in 10 mL of 12 M HCl solution, followed by stirring at 37 °C for 24 h. Subsequently, the suspension underwent centrifugation and was washed until pH > 6. Thereafter, the precipitate was dispersed in 150 mL of deionized water and sonicated at a frequency of 20 kHz and a power of 500 W for 2 h. The dark-green suspension was collected, with the concentration of Ti_3_C_2_T_x_ determined to be 2.0 mg mL^–1^. Finally, the Ti_3_C_2_T_x_ powder was lyophilized and set aside.

### 2.3. Synthesis of Ni_0.5_Co_0.5_Se_2_/Ti_3_C_2_T_x_

**Figure 1:**
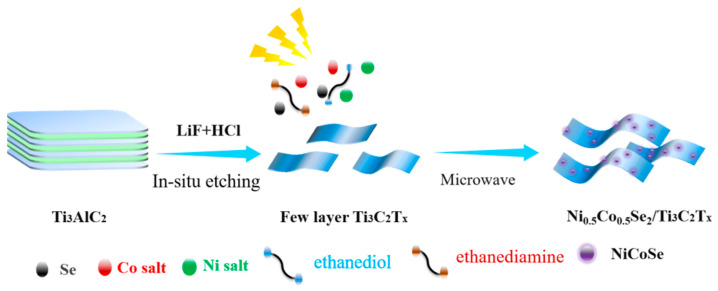
Preparation of Ni_0.5_Co_0.5_Se_2_/Ti_3_C_2_T_x_.

Preparation of Ni_0.5_Co_0.5_Se_2_ preparation steps are shown in Figure 1. The composites were synthesized using a one-step microwave method. First, 40 mg each of Ni(CH_3_COO)_2_·4H_2_O, Co(CH_3_COO)_2_·4H_2_O, and selenium were ground and dispersed in a mixed solution consisting of 800 μL of ethanediamine and 400 μL of ethanediol. Subsequently, 20 mg of Ti_3_C_2_T_x_ powder was added, and the mixture was sonicated at a frequency of 40 kHz and a power of 200 W for 1 h. The resulting homogeneous solution was then transferred to a 900 W microwave oven and irradiated for 120 s. A series of NCSe/Ti_3_C_2_T_x_−*n* (*n* = 1–5) composites were successfully prepared under various conditions shown in Table 1.

### 2.4. Characterization Technique

The crystal structure of the prepared composites was analyzed via X-ray diffraction (XRD) by using an X’Pert Pro diffractometer (PANalytical, Kassel, Germany) with Cu K*α* radiation (2*θ* = 5–80°). The morphology and chemical constitutions of the as-obtained composites were determined via scanning electron microscopy (SEM) by using a TM 4000 microscope (Hitachi, Tokyo, Japan) and transmission electron microscopy (TEM) by using a JEM-F200 multipurpose electron microscope (JEOL Ltd., Tokyo, Japan) equipped with JED-2300T (JEOL Ltd., Tokyo, Japan) for energy-dispersive X-ray spectroscopy EDS. Chemical composition and valence state were further recorded via X-ray photoelectron spectroscopy (XPS) by using the K-Alpha photoelectron spectrometer system (Thermo Fisher Scientific, Waltham, MA, USA), with non-monochromatic Al K*α* X-rays as the excitation source and C1s (284.8 eV) as the reference line for the test analysis.

### 2.5. Electrochemical Measurements

The electrochemical properties of composites were evaluated via cyclic voltammetry (CV), galvanostatic charge–discharge (GCD), and electrochemical impedance spectroscopy (EIS) on an electrochemical workstation (CHI 760E, Shanghai Chenhua Co. Ltd., Shanghai, China) using a three-electrode system in 6 M KOH electrolyte. The working electrode was manufactured by coating the Ni foam (1 × 1 cm^2^, loading mass of 2 mg) with an active material, polyvinylidene fluoride (PVDF), and acetylene black mixed in a mass ratio of 8:1:1. A platinum foil and Hg/HgO in 1 M KOH were used as the counter electrode and reference electrode, respectively. The specific capacitance (Cs) of the electrode was calculated using Equation (1).

Specific capacitance was measured as follows:

(1) $$\mathrm{Cs}=\frac{I∆t}{m∆U}$$

Cs denotes mass specific capacitance (F g^–1^), I denotes current (A), ∆t denotes discharge time (unit: s), m denotes mass of active material (mg), and ∆U denotes voltage window after IR removal (V).

The ASCs were assembled with working electrode as positive electrode, AC as negative electrode, and 3 M KOH solution as electrolyte. The mass ratio of the active materials of the positive electrode (m^+^) and the negative electrode (m^−^) were determined using the following equation:

(2) $$\frac{m^{+}}{m^{–}}=\frac{C^{-}∆V^{-}}{C^{+}∆V^{+}}$$

C^+^ and C^−^ represent the specific capacitance (F g^–1^), while ΔV^+^ and ΔV^−^ represent the discharge voltage (V) of the positive and negative electrodes, respectively.

Energy density equation:

(3) $$E=C\frac{{∆U}^{2}}{7.2}$$

Energy density (W h kg^–1^).

Power density equation:

(4) $$P=3600\frac{E}{∆t}$$

Power density (W kg^–1^).

## 3. Results and Discussion

### 3.1. Synthesis and Characterization

The schematic representation in Figure 1 illustrates the design and synthesis process for the Ni_0.5_Co_0.5_Se_2_/Ti_3_C_2_T_x_ composites. First, the few-layer Ti_3_C_2_T_x_ was obtained via high-power ultrasound after etching with LiF/HCl. Then, nickel salt, cobalt salt, selenium, and the few-layer Ti_3_C_2_T_x_ underwent grinding and dispersion into a mixed solution of ethanediamine and ethanediol. Both selenide and MXene exhibit strong microwave-absorbing properties, allowing for the efficient absorption of microwave energy. The microwave reaction resulted in 3D nanospheres loaded in Ti_3_C_2_T_x_ MXene.

**Figure 2:**
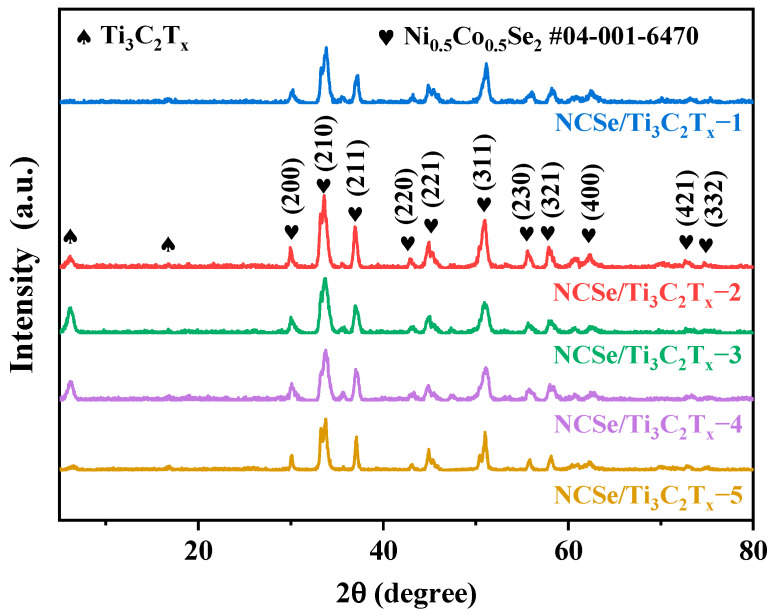
XRD patterns of NCSe/Ti_3_C_2_T_x_−*n* (*n* = 1–5).

XRD was conducted to ascertain the crystal structure of the composites, as shown in Figure 2. The composites exhibited two characteristic diffraction peaks at 6.3° and 16.7°, corresponding to the (002) and (004) crystal plane of the Ti_3_C_2_T_x_ sheets, respectively [23]. The XRD patterns of the composites revealed distinct peaks at approximately 30.3°, 33.9°, 37.4°, 43.2°, 47.7°, 51.1°, 58.4°, 58.5°, 61.9°, 72.9°, and 75.0°, aligning with the (200), (210), (211), (220), (221), (311), (230), (321), (400), (421), and (332) crystal planes of Ni_0.5_Co_0.5_Se_2_ (JCPDS No. 04-001-6470), respectively. No additional impurity peaks were identified, affirming the high purity of the as-obtained composites.

**Figure 3:**
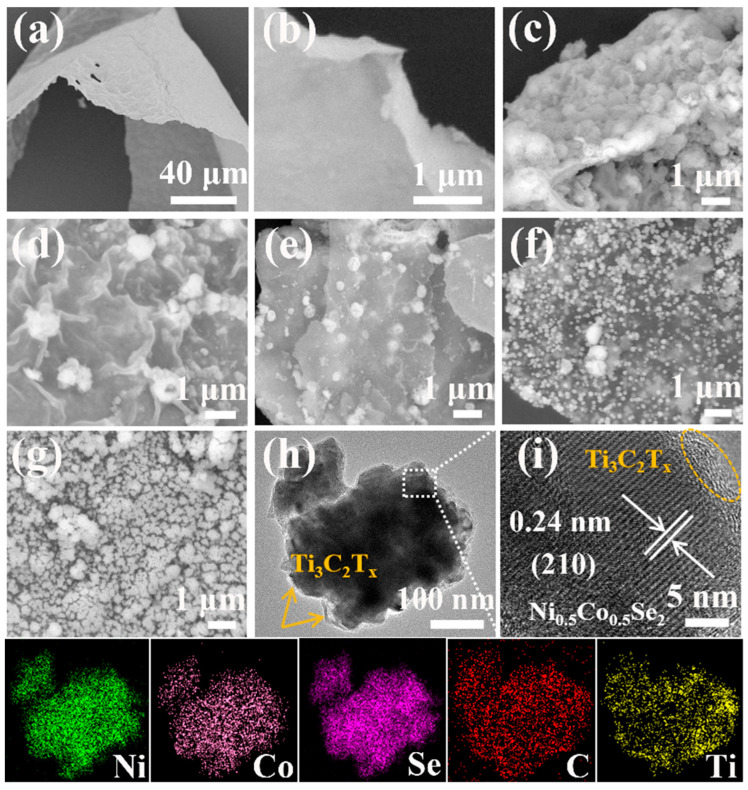
(**a**) Low- and (**b**) high-resolution SEM images of Ti_3_C_2_T_x_. (**c**–**g**) SEM images of composites NCSe/Ti_3_C_2_T_x_−*n* (*n* = 1–5). (**h**) TEM image and corresponding elemental mapping images of NCSe/Ti_3_C_2_T_x_−2. (**i**) HRTEM image.

Figure 3a,b show the lamellar structure of the prepared Ti_3_C_2_T_x_ with a large surface area. Figure 3c–g show the loading of Ni_0.5_Co_0.5_Se_2_ nanospheres within the lamellar Ti_3_C_2_T_x_ structure. In Figure 3c, the composite NCSe/Ti_3_C_2_T_x_−1 obtained with a Ti_3_C_2_T_x_ content of 10 mg shows Ni_0.5_Co_0.5_Se_2_ nanospheres uniformly grown within a few layers of Ti_3_C_2_T_x_ wrapper. In Figure 3d, the Ni_0.5_Co_0.5_Se_2_ nanospheres were uniformly loaded in the few-layer Ti_3_C_2_T_x_ lamellar structure and encapsulated by a monolayer of Ti_3_C_2_T_x_. The Ni_0.5_Co_0.5_Se_2_ nanospheres were loaded in and on the Ti_3_C_2_T_x_, as shown in Figure 3e. This could be attributed to the reduction in the size of the formed Ni_0.5_Co_0.5_Se_2_ nanospheres with increasing amount of Ti_3_C_2_T_x_, leading the composites to a layered structure of Ti_3_C_2_T_x_. In Figure 3d,f,g, with the same Ti_3_C_2_T_x_ addition, the average size of the nanospheres increases with prolonged reaction time. In Figure 3f, when the reaction time was reduced to 90 s, the resultant NCSe/Ti_3_C_2_T_x_−4 exhibits excessively small nanosphere sizes due to inadequate microwave irradiation energy. However, in Figure 3g (sample NCSe/Ti_3_C_2_T_x_−5), excess microwave energy caused the formation of agglomerates.

The morphology of NCSe/Ti_3_C_2_T_x_−2 was further investigated via TEM. From Figure 3h, the Ni_0.5_Co_0.5_Se_2_ nanospheres were located in the Ti_3_C_2_T_x_ wrapper, which is consistent with the results obtained from SEM. The high-resolution (HRTEM) image of the Ni_0.5_Co_0.5_Se_2_ region shows that the crystal plane spacing of Ni_0.5_Co_0.5_Se_2_ is 0.24 nm, which is the same as the plane spacing of Ni_0.5_Co_0.5_Se_2_ (210) [24]. The corresponding energy-dispersive X-ray spectroscopy (EDS) can clearly observe the uniform distribution of nickel, cobalt, selenium, carbon, and titanium throughout the detection area. The aforementioned findings further verified that Ni_0.5_Co_0.5_Se_2_ was successfully grown on the few-layer Ti_3_C_2_T_x_ surface.

**Figure 4:**
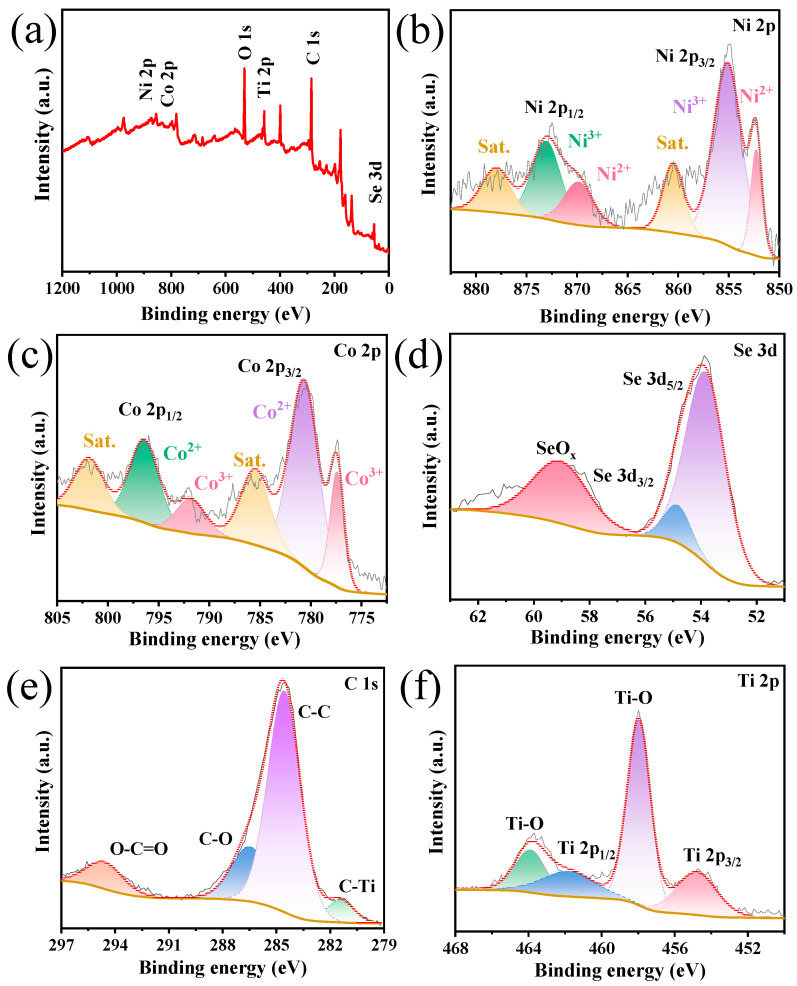
NCSe/Ti_3_C_2_T_x_−2 (**a**) survey XPS, (**b**) Ni 2p, (**c**) Co 2p, (**d**) Se 3d, (**e**) Ti 2p, and (**f**) C 1s.

The surface chemistry and chemical bonding states of NCSe/Ti_3_C_2_T_x_−2 were characterized via XPS. The full spectrum confirms the existence of nickel, cobalt, selenium, carbon, and titanium, consistent with the results from the EDS. In Figure 4c, the Ni 2p spectrum reveals two sets of peaks, corresponding to Ni^3+^ (873 eV and 855.1 eV) and Ni^2+^ (869.8 eV and 852.3 eV), along with satellite peaks (labeled as Sat.) at 878 eV and 860.4 eV [25,26,27]. The Co 2p spectrum (Figure 4c) exhibits peaks related to Co^2+^ (780.7 eV and 796.5 eV) and Co^3+^ (791.7 eV and 777.4 eV) [28]. In Figure 4d, the Se 3d XPS spectrum reveals two peaks at 54.9 eV and 53.9 eV, corresponding to Se 3d_3/2_ and Se 3d_5/2_, respectively [29]. The C 1s spectrum in Figure 4e displays peaks at 294.6, 286.4, 284.6, and 281.4 eV, attributed to the O–C=O, C–O, C–C, and C–Ti bonds, respectively [30]. Figure 4f presents the Ti 2p characteristic peaks of 461.6 eV and 454.8 eV for Ti 2p_1/2_ and Ti 2p_3/2_, respectively [31]. The Ti–O bond at 463.9 eV and 458 eV indicates the formation of oxygen-containing functional groups on the surface of the synthesized composite [32,33]. These experimental results confirm the successful fabrication of NCSe/Ti_3_C_2_T_x_−2.

### 3.2. Electrochemical Measurements

**Figure 5:**
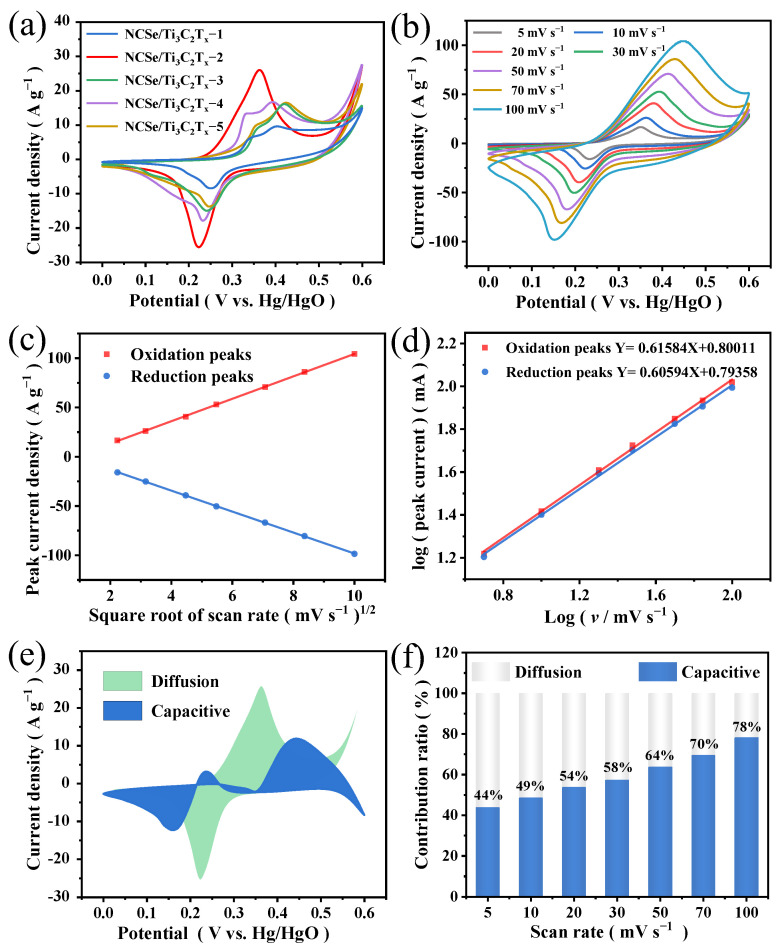
(**a**) CV curves of NCSe/Ti_3_C_2_T_x_−2 electrode materials with different Ti_3_C_2_T_x_ contents at 10 mV s^–1^. (**b**) CV curves of NCSe/Ti_3_C_2_T_x_−2 at different scan rates. (**c**) Relationship of the square root of scan rate and peak current density. (**d**) Plots of log (scan rate) vs. log (peak current) calculated from the CV curves of the NCSe/Ti_3_C_2_T_x_−2 electrode. (**e**) CV profile showing the capacitive and diffusion contributions of NCSe/Ti_3_C_2_T_x_−2 at 10 mV s^–1^. (**f**) Capacitive and diffusion contributions to total capacity at different scan rates.

The electrochemical properties of the supercapacitor electrode materials were investigated in 6 M KOH using a standard three-electrode system. In Figure 5a, the CV curves of NCSe/Ti_3_C_2_T_x_−*n* (*n* = 1–5) at 10 mV s^–1^, the oxidation and reduction peaks largely exist because of Ni^2+^/Ni^3+^ and Co^2+^/Co^3+^ reversible redox reactions [34,35]. The CV curves confirm that the composites have a redox reaction type of battery energy storage. The CV curves of NCSe/Ti_3_C_2_T_x_−2 have a larger integrated area, higher peak current density, and more symmetrical redox peak. These characteristics indicate that NCSe/Ti_3_C_2_T_x_−2 exhibits better electrochemical reversibility. Figure 5b illustrates the CV curves of NCSe/Ti_3_C_2_T_x_−2 acquired under a potential range of 0–0.6 V at different scan rates. The CV curves exhibit a generally symmetrical distribution. As the scanning rate increases, the oxidation peak and the reduction peak shift in opposite directions. This phenomenon is attributed to the growing electrode polarization and internal resistance. Figure 5c demonstrates that the response peak current density exhibits a linear relationship with the square root of the scan rates, implying that the redox reactions are related to the semi-infinite linear diffusion control process. Figure 5d verifies that the slope of the anode peak and cathode peak current density is 0.61584 and 0.60594, respectively. This suggests that the electrochemical reaction occurring at the electrolyte/electrode surface or near surface is reversible. To further explore the charge storage mechanism, the contributions of capacitive capacity and diffusion-controlled capacity are based on the pseudocapacitance formula: i(*V*) = *k*_1_*v* + *k*_2_*v*^1/2^ [36]. Figure 5e shows that the capacitance contribution of the pseudocapacitor of NCSe/Ti_3_C_2_T_x_−2 reaches 49% at 10 mV s^–1^. The high capacitance contribution of NCSe/Ti_3_C_2_T_x_−2 may be attributed to the high conductivity and large surface area of MXene. Furthermore, Figure 5f illustrates that the energy storage mechanism of NCSe/Ti_3_C_2_T_x_−2 is dominated by the capacitance of the pseudocapacitor. The genesis of increasing the contribution of pseudocapacitance primarily relies on the increase in charge transfer rate, electrochemical reaction rate, and surface reaction rate [37].

**Figure 6:**
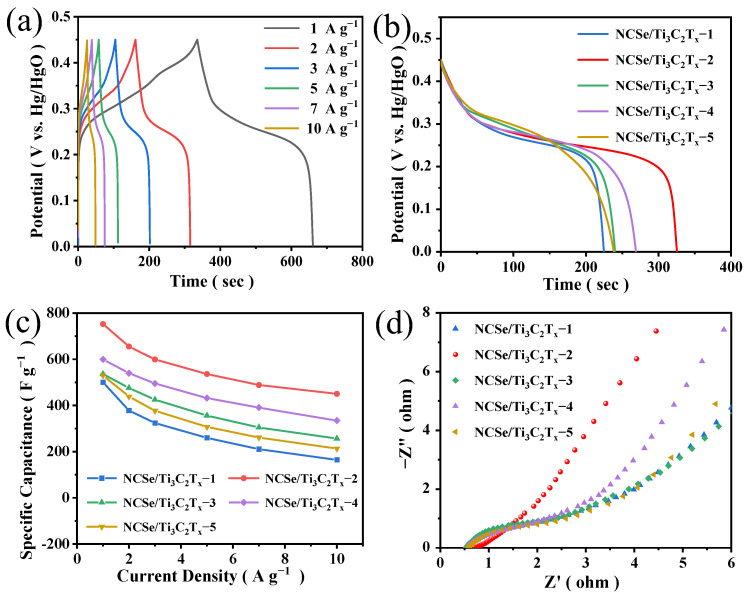
(**a**) GCD curves of NCSe/Ti_3_C_2_T_x_−2 at different current densities, NCSe/Ti_3_C_2_T_x_−*n* (*n* = 1–5). (**b**) GCD curves at a current density of 1 A g^–1^. (**c**) Rate performance with different reaction conditions. (**d**) Nyquist plots.

Figure 6a shows the GCD curves of sample NCSe/Ti_3_C_2_T_x_−2 under different current densities. The GCD curve platform provided by the redox reaction can be observed. Figure 6b illustrates that the addition of appropriate Ti_3_C_2_T_x_ powder can enhance specific capacitance. This enhancement can be attributed to improved microwave absorption, decreased probability of nanospheres agglomeration through an increase in specific surface area, and enhanced conductivity of the composites [28,38]. However, the superfluous powder reduces specific capacitance due to less nanosphere formation during microwave reaction. The results verify that when 20 mg of Ti_3_C_2_T_x_ powder was added, the composite with the optimal specific capacitance (752.4 F g^–1^ at 1 A g^–1^) was obtained. This finding is consistent with the conclusion derived via CV. Capacitance retention rates were calculated based on the capacity variation of different composites at 1–10 A g^–1^ to assess the rate performance at high current densities. In Figure 6c, the capacitance retention rate of NCSe/Ti_3_C_2_T_x_−2 is 60%. Figure 6d presents the EIS plots of the composites, where NCSe/Ti_3_C_2_T_x_−2 still has the smallest semicircle diameter and lower charge transfer resistance. The preceding analysis revealed that NCSe/Ti_3_C_2_T_x_−2 not only has a large capacity but also high electron transfer capability and good capacitance retention rate. This may be attributed to the unique structure of nanospheres uniformly grown in a few layers of Ti_3_C_2_T_x_, promoting efficient electron and ion transport. The disappearance of the semicircular arc in the Nyquist plot of NCSe/Ti_3_C_2_T_x_−2 suggests a distinct behavior compared to other electrodes. This phenomenon stems from its superior reversibility, aligning with the observations from the CV curves. The enhanced reversibility could be attributed to the unique structure of Ni_0.5_Co_0.5_Se_2_ nanospheres, which were uniformly grown within the few-layer Ti_3_C_2_T_x_ wrapper.

**Figure 7:**
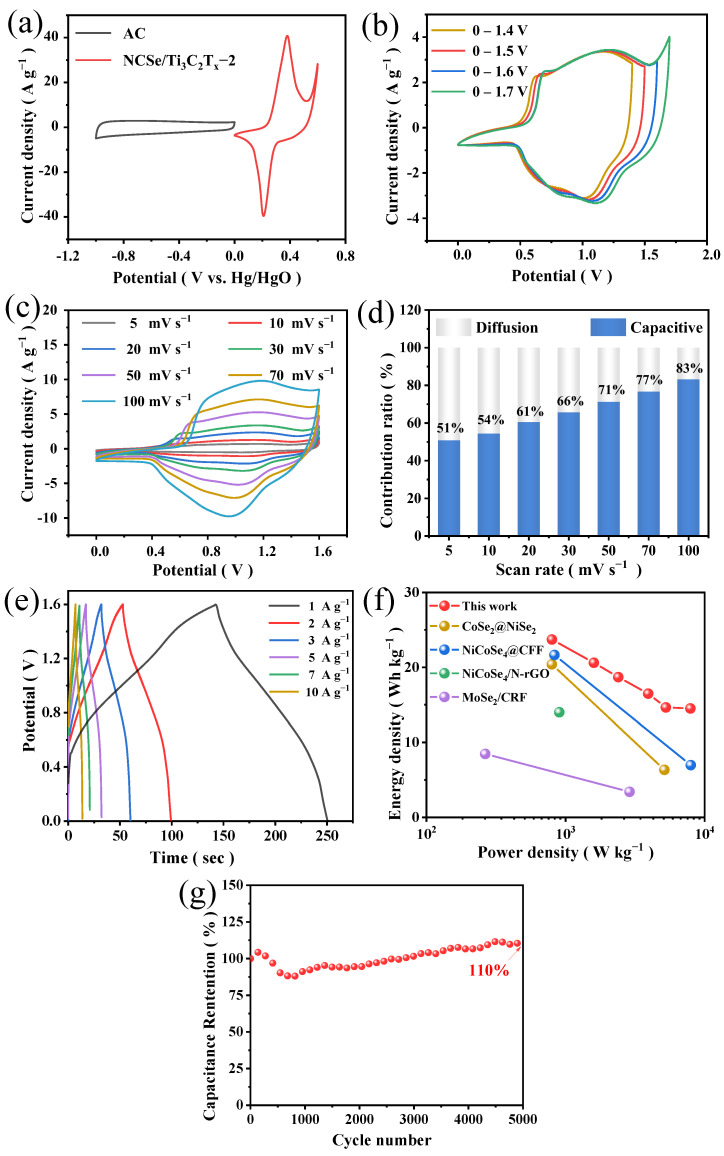
(**a**) CV curves of the AC and NCSe/Ti_3_C_2_T_x_−2 electrodes at 10 mV s^–1^. (**b**) CV curves at different voltage windows (30 m V s^−1^). (**c**) CV curves at different scanning rates. (**d**) Capacitance contribution rate. (**e**) GCD curves of NCSe/Ti_3_C_2_T_x_−2//AC ASC. (**f**) Ragone plots. (**g**) Cyclic life of NCSe/Ti_3_C_2_T_x_−2//AC ASC tested at 3 A g^–1^.

To further explore electrochemical energy storage properties and practicality of NCSe/Ti_3_C_2_T_x_−2, we constructed an asymmetric supercapacitor with 3 M KOH as the electrolyte and AC as the cathode. Based on Equation (2) and charge balance (q^+^ = q^–^), the mass ratio of NCSe/Ti_3_C_2_T_x_−2 and AC electrodes is 0.44 in NCSe/Ti_3_C_2_T_x_−2//AC ASC. To further explore electrochemical energy storage properties and practicality, NCSe/Ti_3_C_2_T_x_−2 was assembled with activated carbon as an asymmetric capacitor for two-electrode tests. Figure 7a shows the CV curves of the positive electrode material NCSe/Ti_3_C_2_T_x_−2 and the negative activated carbon tested at 6 M KOH at a scan rate of 20 mV s^–1^. The working electrodes were battery-type and electrochemical double-layer capacitor EDLC-type materials, with potential windows of 0–0.6 V and −1–0 V, respectively [39,40]. Figure 7b shows that the CV curves were obtained for NCSe/Ti_3_C_2_T_x_−2//AC at 1.4–1.8 V at 30 mV s^−1^. The curves exhibited no significant polarization when the voltage window reached 1.6 V. Therefore, the final operational voltage window of the double electrode was set as 0–1.6 V. In Figure 7c, the CV curves at different scanning rates exhibit good symmetry, indicating that the device has favorable reversibility. In Figure 7d, the contribution rate of the corresponding capacitance gradually increases from 5–100 mV s^−1^. At a scan rate of 5 mV s^−1^, the capacitive contribution accounts for 51%, demonstrating its dominance even at low scan rates.

Figure 7e depicts the GCD curves of NCSe/Ti_3_C_2_T_x_−2//AC at various current densities (1–10 A g^−1^) by calculating the capacities of the composites as 66.82, 58.02, 52.79, 48.02, 46.47, and 41.69 F g^−1^. The conservation rate is 62.4%. The stability aligns with the results obtained from the CV curves. The corresponding Ragone diagram is obtained from the GCD curves. Figure 7f shows that the power density and energy density of the device are 800 W kg^−1^ and 23.7 Wh kg^−1^, respectively. At a high power density of 7.9 KW kg^−1^, the energy density of NCSe/Ti_3_C_2_T_x_−2//AC is maintained at 14.5 Wh kg^−1^, demonstrating better performance than other reported devices, such as CoSe_2_@NiSe_2_ [28], MoSe_2_/CRF [39], NiCoSe_4_@CFF [41], and NiCoSe_4_/N-rGO [42].

Unexpectedly, the stability of NCSe/Ti_3_C_2_T_x_−2//AC was tested at 3A g^–1^. After 5000 cycles, the stability of the device was 110% of the initial value (Figure 7g). This result is likely attributed to the high conductivity of the MXene substrate and the cladding, limiting the volume expansion of selenide. Consequently, surface-active sites were not sufficiently exposed in the beginning of cycling due to the covering of the Ti_3_C_2_T_x_ monolayer film. Therefore, pseudocapacitance rises as cycling progresses after the active sites are fully exposed.

## 4. Conclusions

We synthesized Ni_0.5_Co_0.5_Se_2_ nanospheres loaded within a few-layer Ti_3_C_2_T_x_ structure using an efficient and environmentally friendly microwave-assisted synthesis method. The nanospheres were uniformly distributed, promoting efficient electron transfer and ion migration. The composites exhibited good electrochemical properties, with the NCSe/Ti_3_C_2_T_x_−2 composite electrode achieving a specific capacitance of 752.4 F g^–1^ at 1 A g^–1^. NCSe/Ti_3_C_2_T_x_−2 exhibited the highest capacity and the lowest charge transfer resistance, with remarkable capacitance retention. Impressively, even after 5000 charge/discharge cycles, the capacity retention rate of the NCSe/Ti_3_C_2_T_x_−2//AC asymmetric supercapacitor remained at 110%, indicating its favorable cycling stability. Preparation of composites utilizing the microwave absorption properties of Ti_3_C_2_T_x_ MXene will be promising for applications in the fabrication of supercapacitors with high stability and pseudocapacitive properties.

## Figures and Tables

**Table 1:** A series of NCSe/Ti_3_C_2_T_x_−*n* (*n* = 1–5) composites prepared under various conditions.

Sample	Ti_3_C_2_T_x_ (mg)	Ni(CH_3_COO)_2_·4H_2_O (mg)	Co(CH_3_COO)_2_·4H_2_O (mg)	Se Power (mg)	Microwave Power (W)	Times (s)
NCSe/Ti_3_C_2_T_x_−1	10	40	40	40	900	120
NCSe/Ti_3_C_2_T_x_−2	20	40	40	40	900	120
NCSe/Ti_3_C_2_T_x_−3	30	40	40	40	900	120
NCSe/Ti_3_C_2_T_x_−4	20	40	40	40	900	90
NCSe/Ti_3_C_2_T_x_−5	20	40	40	40	900	150

## Data Availability

Data are contained within the article.

## References

[1] Braff W.A., Mueller J.M., Trancik J.E. (2016). Value of storage technologies for wind and solar energy. Nat. Clim. Chang..

[2] Zhao F., Zheng D., Liu Y., Pan F., Deng Q., Qin C., Li Y., Wang Z. (2021). Flexible Co(OH)_2_/NiO_x_H_y_@Ni hybrid electrodes for high energy density supercapacitors. Chem. Eng. J..

[3] Shang Z., An X., Zhang H., Shen M., Baker F., Liu Y., Liu L., Yang J., Cao H., Xu Q. (2020). Houttuynia-derived nitrogen-doped hierarchically porous carbon for high-performance supercapacitor. Carbon.

[4] Khalafallah D., Quan X., Ouyang C., Zhi M., Hong Z. (2021). Heteroatoms doped porous carbon derived from waste potato peel for supercapacitors. Renew. Energy.

[5] Yue L., Chen L., Liu X., Lu D., Zhou W., Li Y. (2022). Honeycomb-like biomass carbon with planted CoNi3 alloys to form hierarchical composites for high-performance supercapacitors. J. Colloid Interface Sci..

[6] Feng Y., Zhang M., Yan H., Zhang Y., Guo R., Wang H. (2022). Microwave-assisted efficient exfoliation of MXene and its composite for high-performance supercapacitors. Ceram. Int..

[7] Luo L., Zhou Y., Yan W., Du G., Fan M., Zhao W. (2022). Construction of advanced zeolitic imidazolate framework derived cobalt sulfide/MXene composites as high-performance electrodes for supercapacitors. J. Colloid Interface Sci..

[8] Yun X., Lu T., Zhou R., Lu Z., Li J., Zhu Y. (2021). Heterostructured NiSe_2_/CoSe_2_ Hollow Microspheres as Battery-Type Cathode for Hybrid Supercapacitors: Electrochemical Kinetics and Energy Storage Mechanism. Chem. Eng. J..

[9] Yu J., Su H., Shi C., Qiu G., Bai L., Li Z. (2023). Ni0.85Se anchored on N-doped MoSe2 hybrids for long-life asymmetric supercapacitors. Electrochim. Acta.

[10] Arulkumar C., Gandhi R., Vadivel S. (2023). Ultra-thin nanosheets of Ti3C2Tx MXene/MoSe2 nanocomposite electrode for asymmetric supercapacitor and electrocatalytic water splitting. Electrochim. Acta.

[11] Liang T., Lenus S., Liu Y., Chen Y., Sakthivel T., Chen F., Ma F., Dai Z. (2023). Interface and M^3+^/M^2+^ Valence Dual-Engineering on Nickel Cobalt Sulfoselenide/Black Phosphorus Heterostructure for Efficient Water Splitting Electrocatalysis. Energy Environ. Mater..

[12] Lin J., Wang H., Yan Y., Zheng X., Jia H., Qi J., Cao J., Tu J., Fei W., Feng J. (2018). Core-branched CoSe_2_/Ni_0.85_Se nanotube arrays on Ni foam with remarkable electrochemical performance for hybrid supercapacitors. J. Mater. Chem. A..

[13] Zhang Z., Shi Y., Zhao X., Zhou A., Liu R., Che H., Wang G., Mu J., Zhang X., Zhang X. (2022). Construction of hierarchical NiCoSe@CoS core–shell nanotube arrays for high-performance hybrid supercapacitor. J. Alloys Compd..

[14] Jiang H., Wang Z., Yang Q., Tan L., Dong L., Dong M. (2019). Ultrathin Ti_3_C_2_T_x_ (MXene) Nanosheet-Wrapped NiSe_2_ Octahedral Crystal for Enhanced Supercapacitor Performance and Synergetic Electrocatalytic Water Splitting. Nano-Micro Lett..

[15] Wu C.-W., Unnikrishnan B., Chen I.-W.P., Harroun S.G., Chang H.-T., Huang C.-C. (2020). Excellent oxidation resistive MXene aqueous ink for micro-supercapacitor application. Energy Storage Mater..

[16] Wang L., Ma Z., Qiu H., Zhang Y., Yu Z., Gu J. (2022). Significantly Enhanced Electromagnetic Interference Shielding Performances of Epoxy Nanocomposites with Long-Range Aligned Lamellar Structures. Nano-Micro Lett..

[17] Wu N., Zhao B., Chen X., Hou C., Huang M., Alhadhrami A., Mersal G.A.M., Ibrahim M.M., Tian J. (2022). Dielectric properties and electromagnetic simulation of molybdenum disulfide and ferric oxide-modified Ti_3_C_2_T_X_ MXene hetero-structure for potential microwave absorption. Adv. Compos. Hybrid Mater..

[18] Lu Z., Jia F., Zhuo L., Ning D., Gao K., Xie F. (2021). Micro-porous MXene/Aramid nanofibers hybrid aerogel with reversible compression and efficient EMI shielding performance. Compos. Part B Eng..

[19] Zhao K., Sun X., Fu H., Guo H., Wang L., Li D., Liu J. (2023). In situ construction of metal-organic frameworks on chitosan-derived nitrogen self-doped porous carbon for high-performance supercapacitors. J. Colloid Interface Sci..

[20] Yuan Z., Guo H., Huang Y., Li W., Liu Y., Chen K., Yue M., Wang Y. (2022). Composites of NiSe2@C hollow nanospheres wrapped with Ti3C2Tx MXene for synergistic enhanced sodium storage. Chem. Eng. J..

[21] Li Y., Huang B., Zhao X., Luo Z., Liang S., Qin H., Chen L. (2022). Zeolitic imidazolate framework-L-assisted synthesis of inorganic and organic anion-intercalated hetero-trimetallic layered double hydroxide sheets as advanced electrode materials for aqueous asymmetric super-capacitor battery. J. Power Sources.

[22] Sarwar S., Nautiyal A., Cook J., Yuan Y., Li J., Uprety S., Shahbazian-Yassar R., Wang R., Park M., Bozack M.J. (2020). Facile microwave approach towards high performance MoS_2_/graphene nanocomposite for hydrogen evolution reaction. Sci. China Mater..

[23] Dakka Y.A., Balamurugan J., Balaji R., Kim N.H., Lee J.H. (2020). Advanced Cu_0.5_Co_0.5_Se_2_ nanosheets and MXene electrodes for high-performance asymmetric supercapacitors. Chem. Eng. J..

[24] Dong X., Zhao R., Sun B., Zhang T., Wang B., He Y., Gao T., Chao D., Zhou G. (2022). Confining homogeneous Ni_0.5_Co_0.5_Se_2_ nanoparticles in Ti_3_C_2_T_x_ MXene architectures for enhanced sodium storage performance. Appl. Surf. Sci..

[25] Sun X., Zhao K., Fu H., Guo H., Shen J., Jin F., Wang L., Wang Z., Cui L., Quan F. (2023). Heterostructure of MnSe2@NiCo2Se4 as novel electrode material for high-performance asymmetric supercapacitors. J. Energy Storage.

[26] Wang X., Wang S., Chen S., He P., Xu Y., Jia L., Yang D., He X., Deng H., Jia B. (2020). Facile one-pot synthesis of binder-free nano/micro structured dendritic cobalt activated nickel sulfide: A highly efficient electrocatalyst for oxygen evolution reaction. Int. J. Hydrogen Energy.

[27] Adepu V., Kamath K., Mattela V., Sahatiya P. (2021). Development of Ti_3_C_2_T_x_/NiSe_2_ Nanohybrid-Based Large-Area Pressure Sensors as a Smart Bed for Unobtrusive Sleep Monitoring. Adv. Mater. Interfaces.

[28] Wang J., Sarwar S., Song J., Du L., Li T., Zhang Y., Li B., Guo Q., Luo J., Zhang X. (2022). One-step microwave synthesis of self-supported CoSe2@NiSe2 nanoflowers on 3D nickel foam for high performance supercapacitors. J. Alloy. Compd..

[29] Zheng Y., Tian Y., Sarwar S., Luo J., Zhang X. (2020). Carbon nanotubes decorated NiSe2 nanosheets for high-performance supercapacitors. J. Power Sources.

[30] Lu Z., Zhao H., Luo J., Wang J. (2022). Reduced-graphene-oxide-modified self-supported NiSe2 nanospheres on nickel foam as a battery-type electrode material for high-efficiency supercapacitors. J. Phys. Chem. Solids.

[31] Zheng J., Pan X., Huang X., Xiong D., Shang Y., Li X., Wang N., Lau W.-M., Yang H.Y. (2020). Integrated NiCo_2_-LDHs@MXene/rGO aerogel: Componential and structural engineering towards enhanced performance stability of hybrid supercapacitor. Chem. Eng. J..

[32] Tian Y., Yang F., Qiu Z., Jing J., He J., Xu H. (2023). Hierarchical N-Ti_3_C_2_T_X_@NiCo_2_S_4_ Core-Shell Nanosheets Assembled into 3D Porous Hydrogel as Free-Standing Electrodes for High-Performance Supercapacitors. J. Energy Storage.

[33] Wu W., Liu T., Diwu J., Li C., Zhu J. (2023). Metal-Organic Framework–Derived NiCo_2_S_4_@Co_3_S_4_ Yolk-Shell Nanocages/Ti_3_C_2_T_x_ MXene for High-Performance Asymmetric Supercapacitors. J. Alloys Compd..

[34] Ye B., Huang M., Jiang S., Fan L., Lin J., Wu J. (2018). In-situ growth of Se-doped NiTe on nickel foam as positive electrode material for high-performance asymmetric supercapacitor. Mater. Chem. Phys..

[35] Wu S., Cui T., Hu Q., Yin F., Feng Q., Zhou S., Su Q., Wu L., Yang Q. (2020). Mixing solvothermal synthesis nickel selenide on the surface of graphene for high-efficiency asymmetric supercapacitors. Synth. Met..

[36] Augustyn V., Simon P., Dunn B. (2014). Pseudocapacitive oxide materials for high-rate electrochemical energy storage. Energy Environ. Sci..

[37] Pathak M., Tamang D., Kandasamy M., Chakraborty B., Rout C.S. (2020). A comparative experimental and theoretical investigation on energy storage performance of CoSe_2_, NiSe_2_ and MnSe_2_ nanostructures. Appl. Mater. Today.

[38] Liu J., Ren L., Luo J., Song J. (2022). Microwave synthesis of NiSe/NiTe2 nanocomposite grown in situ on Ni foam for all-solid-state asymmetric supercapacitors. Colloids Surfaces A Physicochem. Eng. Asp..

[39] Han W., Yuan L., Liu X., Wang C., Li J. (2021). Ultrathin MoSe2 nanosheets decorated on carbon aerogel microspheres for high-capacity supercapacitor electrodes. J. Electroanal. Chem..

[40] Bo X., Xiang K., Zhang Y., Shen Y., Chen S., Wang Y., Xie M., Guo X. (2019). Microwave-assisted conversion of biomass wastes to pseudocapacitive mesoporous carbon for high-performance supercapacitor. J. Energy Chem..

[41] Du L., Lv N., Li J., Zhang J., Chen Y., Zhang Y., Li Z., Huang X., Luo J. (2023). NiCoSe_4_@CFF with excellent properties prepared by microwave method for flexible supercapacitors and oxygen evolution reaction. J. Ind. Eng. Chem..

[42] Yang Q., Feng Q., Xu X., Liu Y., Yang X., Yang F., Li J., Zhan H., Wang Q., Wu S. (2022). NiCoSe_4_ nanoparticles derived from nickel–cobalt Prussian blue analogues on N-doped reduced graphene oxide for high-performance asymmetric supercapacitors. Nanotechnology.

